# Combination of PI-RADS score and PSAD can improve the diagnostic accuracy of prostate cancer and reduce unnecessary prostate biopsies

**DOI:** 10.3389/fonc.2022.1024204

**Published:** 2022-11-16

**Authors:** Changming Wang, Lei Yuan, Deyun Shen, Bin Zhang, Baorui Wu, Panrui Zhang, Jun Xiao, Tao Tao

**Affiliations:** ^1^ Department of Urology, The First Affiliated Hospital of USTC of China, Division of Life Sciences and Medicine, University of Science and Technology of China, Hefei, China; ^2^ Department of Radiology, The First Affiliated Hospital of USTC, Division of Life Sciences and Medicine, University of Science and Technology of China, Hefei, China; ^3^ Department of Urology, Affiliated Anhui Provincial Hospital of Anhui Medical University, Hefei, China; ^4^ Hefei National Laboratory for Physical Sciences at Microscale, The CAS Key Laboratory of Innate Immunity and Chronic Disease, School of Basic Medical Sciences, Division of Life Sciences and Medicine, University of Science and Technology of China, Hefei, China

**Keywords:** prostate cancer, prostate biopsy, multiparameter magnetic resonance imaging, prostate imaging-reporting and data system score, prostate-specific antigen density

## Abstract

**Objectives:**

The purpose of this study is to evaluate the diagnostic accuracy of the clinical variables of patients with prostate cancer (PCa) and to provide a strategy to reduce unnecessary biopsies.

**Patients and methods:**

A Chinese cohort that consists of 833 consecutive patients who underwent prostate biopsies from January 2018 to April 2022 was collected in this retrospective study. Diagnostic ability for total PCa and clinically significant PCa (csPCa) was evaluated by prostate imaging–reporting and data system (PI-RADS) score and other clinical variables. Univariate and multivariable logistic regression analyses were performed to figure out the independent predictors. Diagnostic accuracy was estimated by plotting receiver operating characteristic curves.

**Results:**

The results of univariate and multivariable analyses demonstrated that the PI-RADS score (P < 0.001, OR: 5.724, 95% CI: 4.517–7.253)/(P < 0.001, OR: 5.199, 95% CI: 4.039–6.488) and prostate-specific antigen density (PSAD) (P < 0.001, OR: 2.756, 95% CI: 1.560–4.870)/(P < 0.001, OR: 4.726, 95% CI: 2.661–8.396) were the independent clinical factors for predicting total PCa/csPCa. The combination of the PI-RADS score and PSAD presented the best diagnostic performance for the detection of PCa and csPCa. For the diagnostic criterion of “PI-RADS score ≥ 3 or PSAD ≥ 0.3”, the sensitivity and negative predictive values were 94.0% and 93.1% for the diagnosis of total PCa and 99.2% and 99.3% for the diagnosis of csPCa, respectively. For the diagnostic criterion “PI-RADS score >3 and PSAD ≥ 0.3”, the specificity and positive predictive values were 96.8% and 92.6% for the diagnosis of total PCa and 93.5% and 82.4% for the diagnosis of csPCa, respectively.

**Conclusions:**

The combination of the PI-RADS score and PSAD can implement the extraordinary diagnostic performance of PCa. Many patients may safely execute active surveillance or take systematic treatment without prostate biopsies by stratification according to the PI-RADS score and the value of PSAD.

## Introduction

Prostate cancer (PCa) is the most common malignancy of the male genitourinary system. According to the latest data, there will be 268,490 new diagnosed cases and 34,500 deaths in the United States in 2022 (1). In China, with the rapid development of economy and wide adoption of early detection techniques, the incidence of PCa is gradually increasing year by year (2). The incidence of PCa is closely related to the age of the patients; a study has shown that PCa is extremely rare in men under 50 years of age, but more than 85% of the patients are over 60 years of age (3). Therefore, the increasing aging of the Chinese population will inevitably lead to a fast increase in the number of patients with PCa. In the face of the rapidly growing patient population, early screening, diagnosis, and treatment of PCa have great clinical significance to improve prognosis, reduce the proportion of advanced cases, and prolong life span (4).

To date, the main methods recommended by the guidelines for the early detection of PCa include digital rectal examination (DRE), serum total prostate-specific antigen (tPSA), transrectal ultrasound, and genetic tests for inherited PCa (5). Results of DRE by different operators were inconsistent, and both the pooled sensitivity and specificity are less than 60% (6). Serum tPSA has satisfactory sensitivity for the diagnosis of PCa, but elevated PSA is not specific for PCa; some PSA derivatives, such as PSA density (PSAD), PSA velocity, PSA doubling time, and free/total PSA ratio, also have a fairly diagnostic value for PCa, but their clinical value is still controversial, and more high-quality studies are still necessary before clinical practice (7, 8). Multiparameter magnetic resonance imaging (mpMRI) has been widely used in the diagnosis of PCa in recent years. The results of mpMRI can be quantitatively evaluated by prostate imaging–reporting and data system (PI-RADS) (9). A study found that the addition of PSAD can improve the predictive performance of PI-RADS for the identification of PCa (10). However, mpMRI has poor identification of small masses, inflammatory lesions, and low-grade PCa (11).

Ultimately, prostate biopsies are required to confirm the diagnosis of suspected patients. Although prostate biopsy is the current gold standard for diagnosing PCa, it still has some deficiencies such as unpredictable complications, and most important is that the detection rate of PCa by prostate biopsy is less than 50% in light of the previous studies (12, 13). The purpose of this study was to evaluate the diagnostic accuracy of mpMRI and clinical parameters and to propose a strategy to reduce unnecessary prostate biopsies.

## Patients and methods

### Patients and selection criteria

This study was approved by the ethics committee of The First Affiliated Hospital of USTC (No. 2022-RE-125). In total, the information of 833 consecutive patients who accepted prostate biopsies from January 2018 to April 2022 was collected from the Department of Urology at The First Affiliated Hospital of USTC to accomplish this retrospective analysis. The baseline clinicopathologic features of all the patients were obtained by the methods that we described previously (14). Only patients with naïve prostate biopsy and complete clinicopathologic characteristics could be included in this study; meanwhile, patients were still excluded for any of the following conditions: a history of other malignancies, more than 2 weeks from laboratory tests to operations, have taken 5a-reductase inhibitors before biopsy, and serum tPSA ≥100 or <4 ng/ml. Each participant signed an informed consent prior to biopsy.

### MRI image acquisition and PI-RADS score

All enrolled patients underwent mpMRI examinations with a 3.0T scanner with an external six-channel body array coil (Trio Tim, Siemens Healthineers, Erlangen, Germany). Patients were placed in the supine position, and endorectal coils were not used. The imaging protocol included transverse T1-weighted imaging (T1WI), multiplanar (transverse, sagittal, and coronal) T2-weighted imaging (T2WI), and transverse diffusion-weighted imaging (DWI) with a quantitative apparent diffusion coefficient (ADC) (*b* values were 0, 800, and 1,400 s/mm^2^). All the images required within 2 months before prostate biopsy. Then, the interpretation was performed by two professional radiologists with more than 3 years of experience in prostate mpMRI. They first reviewed the images separately and discussed the controversial results together subsequently. They were blinded to the pathological results throughout the process. Ultimately, a definite PI-RADS score (version 2.1) from 1 to 5 was obtained for every incorporated participant (15). **Figure S1** shows the representative images of mpMRI.

### Biopsy protocol and histopathological results

In our hospital, prostate biopsies were performed by specialized urologists; all patients underwent transperineal procedures with the help of a transrectal ultrasound-guided system (biplane imaging scan). Systematic biopsy with a 12-core protocol was performed for each patient at first, and patients who had regions of interest in mpMRI (PI-RADS score ≥ 3) would receive cognitive fusion–targeted biopsy with additional one to six cores. All samples were sent to the pathology department for standard histological examinations, which was also regarded as the “gold standard” in this study. Histopathological grade was recorded according to the International Society of Urological Pathology 2014 updated Gleason score grading system (16). The primary endpoint of our study was the detection rate of clinically significant PCa (csPCa) defined as high-grade PCa with Gleason score ≥ 3 + 4, and clinically insignificant PCa (cisPCa) refers to low-grade PCa with Gleason score = 3 + 3.

### Statistical analyses

Non-normal distributed continuous variables were presented as median [interquartile ranges (IQRs)] and compared by the Kruskal–Wallis test. Descriptive statistic counts (proportions) and chi-square test were used to describe the categorical variables. The correlation coefficients were evaluated using Spearman’s rank correlation analysis. Univariate and multivariable logistic regression analyses were applied to screen the independent predictors of PCa or csPCa, and the odds ratio (OR) and 95% confidence interval (95% CI) were also recorded. Diagnostic performance was evaluated by plotting receiver operating characteristic (ROC) curves and their values of area under the curve (AUC). Sensitivity and specificity were calculated for clinical variables and the probability of combined PI-RADS score with PSAD at the optimal cutoff value. The accuracy of diagnostic tests was evaluated by sensitivity, specificity, positive predictive values (PPVs), and negative predictive values (NPVs) for different diagnostic criteria. Statistical analysis was performed using IBM SPSS (version 25.0) and R software (version 4.2.0) (http://www.R-project.org), and ROC curves were plotted and compared using MedCalc (version 18.9.1). P < 0.05 was considered statistically significant.

## Results

### Demographic characteristics of the enrolled patients

**Figure 1:**
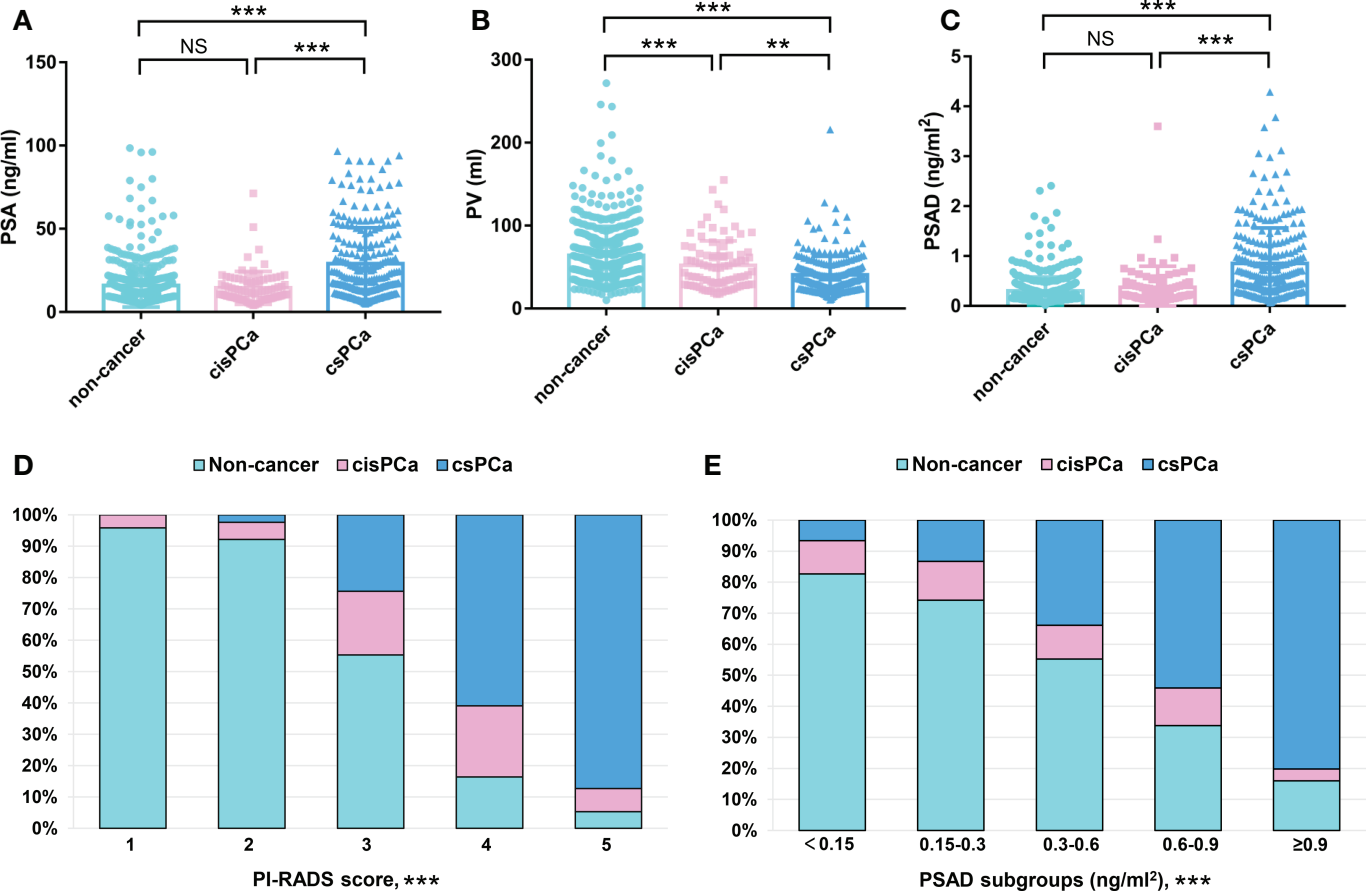
Comparisons of the clinical variables among the non-cancer, csPCa, and cisPCa patients: **(A)** total PSA; **(B)** PV; **(C)** PSAD; **(D)** PI-RADS score; **(E)** PSAD subgroups. **P < 0.01; ***P < 0.001; NS, not significant.

The original information of all the patients was summarized in **Table S1**. In total, 833 patients were incorporated in the retrospective analysis; there were 336/833 (40.3%) PCa cases and 497/833 (59.7%) cases with a non-cancerous outcome. Within these patients with PCa, 248/336 (73.8%) were diagnosed with csPCa, and 88/336 (26.2%) were diagnosed with cisPCa. The median value (IQR) of age, body mass index (BMI), PSA, prostate volume (PV), and PSAD were 69 (63–75) years, 23.77 (21.80–25.50) kg/m^2^, 13.83 (9.36–21.61) ng/ml, 47.62 (32.18–67.78) ml, and 0.30 (0.16–0.52) ng/ml^2^ of all the patients, respectively. Comparisons of these clinical variables among the non-cancer, csPCa, and cisPCa patients revealed that PSA and PSAD levels were significantly higher in the csPCa group (P < 0.001) (**Figures 1A, C**). The PV of the non-cancer group was the biggest followed by the cisPCa and csPCa groups (P < 0.01) (**Figure 1B**). After stratifying patients by the PI-RADS score and PSAD subgroups, the detection rate of csPCa increased dramatically with an elevated PSAD level and PI-RADS score (P < 0.001) (**Figures 1D, E**). These data discovered that the PI-RADS score and PSAD have potential discriminative ability for prostate biopsy results.

### PI-RADS score and PSAD were independent predictors of the prostate biopsy results

First, correlation analysis indicated that the PI-RADS score and PSAD were two main factors related to PCa and csPCa detection (**Figure S2**). Then, the results of univariate analysis revealed that age, PSA, PV, PSAD, and PI-RADS score were associated factors for both PCa and csPCa. Because PSAD had strong correlations with PSA and PV (**Figure S2**), PSA and PV were excluded from the multivariable analysis to avoid confounding. The results of multivariable analysis demonstrated that the PI-RADS score (P < 0.001, OR: 5.724, 95% CI: 4.517–7.253; P < 0.001, OR: 5.199, 95% CI: 4.039–6.488) and PSAD (P < 0.001, OR: 2.756, 95% CI: 1.560–4.870; P < 0.001, OR: 4.726, 95% CI: 2.661–8.396) were independent clinical factors to predict PCa and csPCa, respectively. The detailed data of univariate and multivariable analyses were concluded in **Table 1**.

**Table 1:** Univariate and multivariable analysis for screening out the independent factors of total PCa and csPCa.

Parameters	Univariate analysis			Multivariable analysis			
	OR	95% CI	P-value	B	OR	95% CI	P-value
For total PCa							
Age (years)	1.059	1.041–1.078	<0.001	0.024	1.024	0.999-1.051	0.064
BMI (kg/m^2^)	1.000	0.954–1.049	0.991				
PSA (ng/ml)	1.037	1.026–1.047	<0.001				
PV (ml)	0.973	0.967–0.979	<0.001				
PSAD (ng/ml^2^)	12.154	7.303–20.227	<0.001	1.014	2.756	1.560–4.870	<0.001
PI-RADS score	6.551	5.214–8.231	<0.001	1.745	5.724	4.517–7.253	<0.001
For csPCa							
Age (years)	1.063	1.043–1.084	<0.001	0.028	1.028	0.999–1.058	0.055
BMI (kg/m^2^)	0.977	0.928–1.028	0.375				
PSA (ng/ml)	1.049	1.038–1.060	<0.001				
PV (ml)	0.967	0.959–0.974	<0.001				
PSAD (ng/ml^2^)	15.438	9.329–25.545	<0.001	1.553	4.726	2.661–8.396	<0.001
PI-RADS score	6.199	4.940–7.779	<0.001	1.633	5.199	4.039–6.488	<0.001

PCa, prostate cancer; csPCa, clinically significant prostate cancer; BMI, body mass index; PSA, prostate-specific antigen; PV, prostate volume; PSAD, prostate-specific antigen density; PI-RADS, prostate imaging-reporting and data system; OR, odds ratio; 95% CI, 95% confidence interval.

### Diagnostic performance of the clinical variables and combined PI-RADS score with PSAD

**Figure 2:**
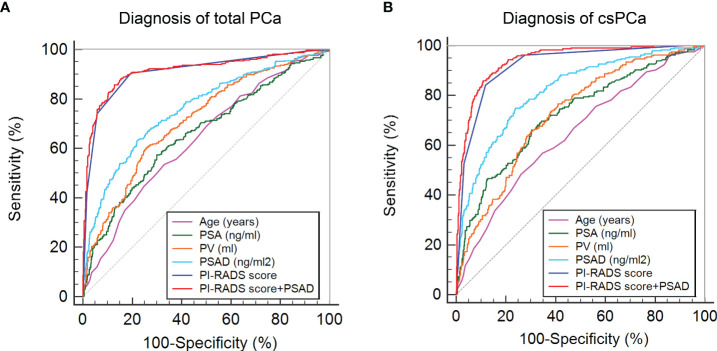
ROC curves of clinical variables and combined PI-RADS score with PSAD for the diagnosis of PCa: **(A)** ROC curves for the diagnosis of total PCa; **(B)** ROC curves for the diagnosis of csPCa.

First, the PI-RADS score and PSAD were combined according to the results of multivariable analysis. By plotting ROC curves, the combination of the PI-RADS score and PSAD presented with the best diagnostic accuracy for PCa (**Figure 2A**) and csPCa (**Figure 2B**) in prostate biopsy compared with any single clinical variable. Regarding the diagnosis of PCa, the AUC, sensitivity, and specificity were 0.915 (95% CI: 0.894–0.933), 85.71%, and 87.12%, respectively, for the combined PI-RADS score with PSAD, which was obviously higher than age, PSA, PV, and PSAD (P < 0.001), but no statistical difference was observed compared with the PI-RADS score (P = 0.148) (**Table 2**). In terms of the csPCa diagnosis, the AUC, sensitivity, and specificity were 0.942 (95% CI: 0.924–0.957), 85.89%, and 89.06%, respectively, for the combined PI-RADS score with PSAD; the diagnostic accuracy significantly outperformed any single clinical variable including the PI-RADS score (P < 0.001) (**Table 2**). Above all, the combination of the PI-RADS score and PSAD received the best diagnostic performance for the detection of PCa and csPCa in prostate biopsy.

**Table 2:** Diagnostic accuracy of the clinical parameters for total PCa and csPCa.

Parameters	AUC	95% CI	Sensitivity	Specificity	P-value
For total PCa					
Age (years)	0.637	0.603-0.670	53.47%	67.00%	<0.001
PSA (ng/ml)	0.660	0.627-0.692	57.44%	70.02%	<0.001
PV (ml)	0.713	0.681-0.744	60.12%	74.45%	<0.001
PSAD (ng/ml^2^)	0.760	0.729-0.788	63.69%	77.46%	<0.001
PI-RADS score	0.909	0.887-0.928	90.48%	80.48%	0.148
PI-RADS score + PSAD	0.915	0.894-0.933	85.71%	87.12%	Reference
For csPCa					
Age (years)	0.645	0.612-0.678	48.39%	73.85%	<0.001
PSA (ng/ml)	0.723	0.692-0.753	66.13%	69.57%	<0.001
PV (ml)	0.726	0.694-0.756	76.61%	58.97%	<0.001
PSAD (ng/ml^2^)	0.820	0.792-0.845	75.00%	76.07%	<0.001
PI-RADS score	0.922	0.902-0.939	84.27%	88.21%	<0.001
PI-RADS score + PSAD	0.942	0.924-0.957	85.89%	89.06%	Reference

PCa, prostate cancer; PSA, prostate-specific antigen; PV, prostate volume; PSAD, prostate-specific antigen density; PI-RADS, prostate imaging-reporting and data system; csPCa, clinically significant prostate cancer; AUC, area under curve; 95% CI, 95% confidence interval.

### PCa and csPCa detection rate in patients stratified by PI-RADS score and PSAD

**Figure 3:**
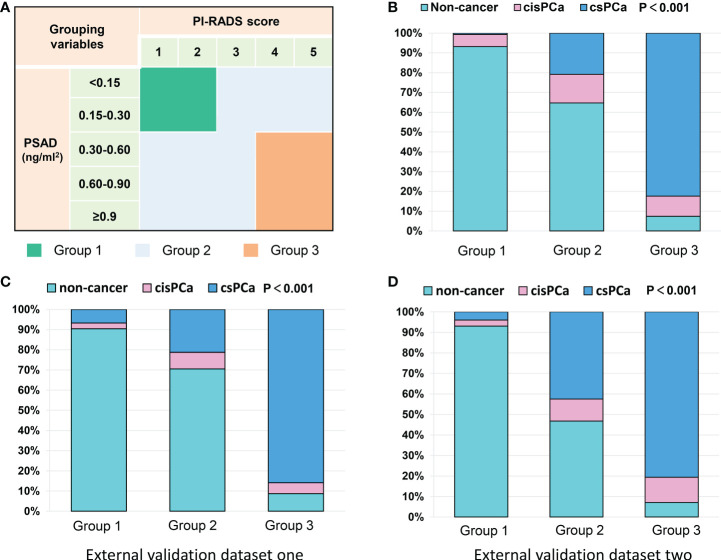
Grouping scheme of patients and frequency distribution in each group: **(A)** grouping scheme of patients by PI-RADS score and PSAD subgroups; **(B)** frequency distribution of the patients in different groups; **(C, D)** frequency distribution histograms of two external datasets.

To counsel patients for reducing unnecessary prostate biopsies, an exact diagnostic threshold value of the PI-RADS score and PSAD is needed. Then, all patients were divided into different groups according to the separated PI-RADS score and PSAD subgroups. Subsequently, we calculated the detection rates of total PCa and csPCa in these groups, which are exhibited in **Tables S2** and **S3**, respectively. After careful consideration, 291 patients with “PI-RADS score < 3 and PSAD < 0.3” were categorized into group 1; 326 patients with “PI-RADS score ≥ 3 and PSAD < 0.3” or “PI-RADS score < 3 and PSAD ≥ 0.3” were regarded as group 2; and 216 patients with “PI-RADS score > 3 and PSAD ≥ 0.3” were defined as group 3 (**Figure 3A**). The distribution and frequencies of the patients in each group are summarized in **Table S4**. We found only 2/291 (0.7%) patients diagnosed with csPCa in group 1 and only 16/216 (7.4%) patients diagnosed with non-PCa by prostate biopsy in group 3 (**Figure 3B**). Therefore, we established two diagnostic criteria: criterion 1 is “PI-RADS scored ≥ 3 or PSAD ≥ 0.3”, and the sensitivity, specificity, PPV, and NPV of criterion 1 were 94.0%, 54.5%, 58.3%, and 93.1% for the diagnosis of total PCa and 99.2%, 49.4%, 45.4%, and 99.3% for the diagnosis of csPCa, respectively; criterion 2 is “PI-RADS score > 3 and PSAD ≥ 0.3”, and the sensitivity, specificity, PPV, and NPV of criterion 2 were 59.5%, 96.8%, 92.6%, and 78.0% for the diagnosis of total PCa and 71.8%, 93.5%, 82.4%, and 88.7% for the diagnosis of csPCa, respectively (**Table 3**). These data suggest that patients with negative results by diagnostic criterion 1 can almost rule out the possibility of PCa and, inversely, a high probability of PCa for patients with positive results by diagnostic criterion 2.

**Table 3:** Diagnostic accuracy of total PCa and csPCa by different diagnostic threshold.

Diagnostic threshold	Sensitivity	Specificity	PPV	NPV	Accuracy
For total PCa					
PI-RADS scored ≥ 3 or PSAD ≥ 0.3	94.0%	54.5%	58.3%	93.1%	70.5%
PI-RADS score >3 and PSAD ≥ 0.3	59.5%	96.8%	92.6%	78.0%	81.8%
For csPCa					
PI-RADS scored ≥ 3 or PSAD ≥ 0.3	99.2%	49.4%	45.4%	99.3%	64.2%
PI-RADS score >3 and PSAD ≥ 0.3	71.8%	93.5%	82.4%	88.7%	87.0%

PCa, prostate cancer; csPCa, clinically significant prostate cancer; PI-RADS, prostate imaging-reporting and data system; PSAD, prostate-specific antigen density; PPV, positive predictive value; NPV, negative predictive value.

### External validation of our results by other Chinese datasets

Our results were also externally validated in two other Chinese datasets from the recent report by Tao et al. (14). As we expected, after the patients were categorized into three groups by the PI-RADS score and PSAD with the method mentioned in **Figure 3A**, the frequency distribution histograms indicated significant discrepancies of patients’ composition (**Figures 3C, D**). In the external validation dataset 1, only 7/104 (6.7%) patients were diagnosed with csPCa in group 1, and just 8/92 (8.7%) patients were excluded from diagnosis of PCa in group 3. Similarly, in the second external validation dataset, only 4/101 (3.9%) patients were diagnosed with csPCa in group 1, and just 7/98 (9.1%) patients were diagnosed with non-cancerous diseases in group 3 (**Table S4**). These data illustrated a pretty good performance of multicenter verifications.

### Strategy for avoiding unnecessary prostate biopsy

**Figure 4:**
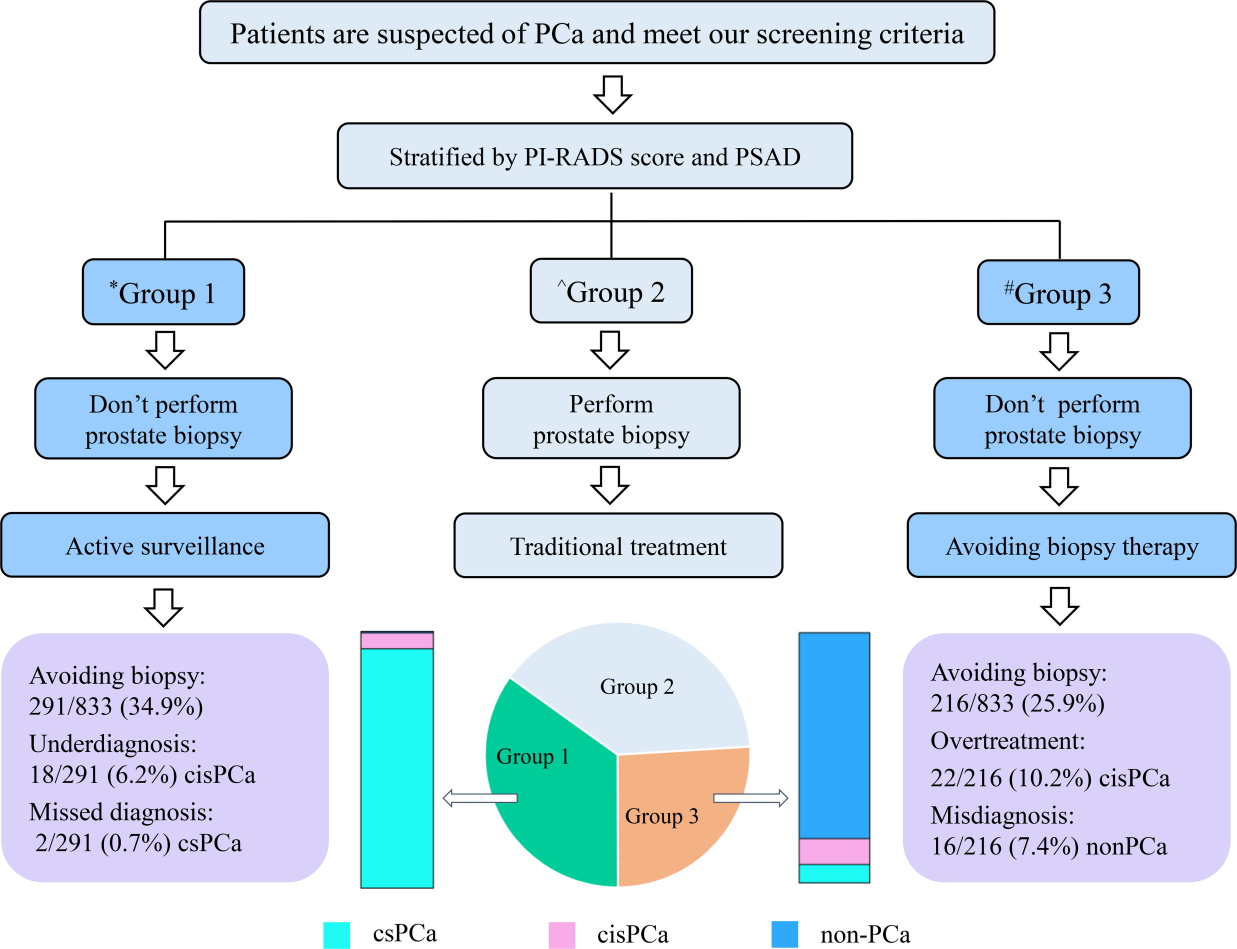
Flowchart of the strategy for avoiding unnecessary prostate biopsy. ^*^Group 1 patients with PI-RADS score < 3 and PSAD < 0.3; ^^^Group 2 patients with “PI-RADS score ≥ 3 and PSAD < 0.3” or “PI-RADS score < 3 and PSAD ≥ 0.3”; ^#^Group 3 patients with PI-RADS score > 3 and PSAD ≥ 0.3.

Finally, a strategy was established to avoid unnecessary prostate biopsies (**Figure 4**). Outpatients with suspicion of PCa can be stratified by the combination of the PI-RADS score and PSAD, and patients categorized into group 2 should accept routine prostate biopsies; patients divided into group 1 can safely avoid biopsies and carry out active surveillance on account of only 2/291 (0.7%) csPCa cases that received missed diagnoses in the current observation. In addition, patients in group 3 can also take radical or systemic therapy without prostate biopsies into consideration because only 16/216 (7.4%) patients with non-PCa were observed in group 3 of this study. However, this may still be full of challenges because of the irretrievable destruction by radical prostatectomy for patients without PCa even if they are in group 3.

## Discussion

In recent decades, prostate biopsy has been the most commonly recommended method for the early diagnosis of PCa, but it has some unavoidable shortcomings. First of all, prostate biopsy is invasive and can cause postoperative complications such as sepsis and bleeding (17). Second, the operation will result in a certain degree of psychological and financial burden to patients. Then, for the patients diagnosed with csPCa by biopsies, they need to wait a period of time before radical prostatectomy, and this will increase the probability of cancer dissemination. Furthermore, because of the possibility of false-negative biopsy results, some patients have to undergo repeated biopsies (18). At last, in many studies, the detection rate of PCa or csPCa is less than 50%, which means a general phenomenon that lots of patients accepted undue biopsies (19). Our purpose in this retrospective study is to propose a strategy for clinicians to obviate needless prostate biopsies.

Serum tPSA examination is the most commonly used tool for PCa screening. Abnormal non-specific escalation of tPSA is the primary reason of the unnecessary prostate biopsies (20). To make a triage test of patients prior to biopsy, some risk calculators that incorporated tPSA have been established, such as Prostate Cancer Prevention Trial Risk Calculator (21) and European Randomized Study of Screening for Prostate Cancer Risk Calculator (22); however, studies have proved that it will lead to overdiagnosis and overtreatment when these calculators are applied in the Chinese populations, and, as a result, the median or average level of tPSA is usually higher in Chinese patients compared with that in Western cohorts (23, 24). Importantly, the value of mpMRI was not estimated in these studies. mpMRI is a routine examination recommended prior to biopsy nowadays, and latest meta-analyses indicated that the pooled NPV of mpMRI using the definition of negative MRI (PI-RADS score 1–2) and csPCa (Gleason score ≥ 3 + 4 = 7) was 90.8% (95% CI: 88.1–93.1%) for biopsy-naïve men (25). However, the pooled PPV of suspicious mpMRI for csPCa was only 42% (95% CI: 38–45%) in the biopsy-naïve group (26). MRI-guided targeted biopsy can enhance the detection of csPCa and detect significantly fewer cisPCa than systematic biopsy (27). PSAD is the value of serum tPSA divided by the PV, and previous studies have demonstrated that mpMRI combined with PSAD < 0.15 ng/ml^2^ can improve the NPV to predict PCa (28, 29). However, these studies were based on Western populations with a small sample size, and there is still a paucity in the data from Chinese patients.

In the present study, we observed the diagnostic value of patients’ clinical variables and found that PSAD and the PI-RADS score can independently predict PCa and csPCa of prostate biopsies. The combination of PSAD and the PI-RADS score achieved the best diagnostic performance relative to using a single variable. By setting different diagnostic criteria, we discovered that patients with “PI-RADS score < 3 and PSAD < 0.3” can safely rule out the diagnosis of csPCa, and we make a definitive diagnosis of PCa for patients with “PI-RADS score > 3 and PSAD ≥ 0.3”. To reduce unnecessary prostate biopsies, most of the previous studies discussed the diagnostic threshold with a high NPV, just like the diagnostic criterion 1 that we described above. However, we also discussed a diagnostic criterion with a high PPV. Radical prostatectomy without biopsy is a viable option despite 16/216 (7.4%) patients with PI-RADS score > 3 and PSAD ≥ 0.3 diagnosed with non-cancerous diseases in this study. On the one hand, prostate biopsies could produce false-negative results. In addition, ^68^Ga prostate-specific membrane antigen positron emission tomography/computed tomography (^68^Ga PSMA PET/CT) is a novel diagnostic modality with excellent performance for both primary and metastatic lesions of PCa (30). A recent study has reported that men with PI-RADS of 4 or 5 combined with a maximum standardized uptake value (SUVmax) ≥ 9 can denote csPCa with 100% specificity (31). In addition, the initial successful experience has been released for 25 patients who received radical prostatectomy without prior biopsy; all these patients got PI-RADS score ≥ 4 in mpMRI and SUVmax ≥ 4.0 in ^68^Ga PSMA PET/CT (32). In the future, for patients with high suspicion of PCa, prostate biopsy may no longer be the only way for diagnosis before active therapies.

Our study also has some limitations. First, the suspicious regions in the mpMRI with a PI-RADS score of 4 or 5 were detected by cognitive fusion–targeted biopsies, which can produce inevitable deviation without a real-time intraoperative MRI-guided system. Second, some important clinical parameters like DRE and free/total PSA were not analyzed because of too many irretrievable missing values. Third, although mpMRI images were independently reviewed by two radiologists, inter-observer reliability was not assessed. Next, this study was only validated in few tertiary medical centers, and it should be validated in other Chinese high-volume hospitals in the future. Last, selection bias cannot be avoided due to the retrospective nature.

## Conclusions

Prostate biopsy is the most commonly used approach for the initial diagnosis of PCa with several inherent shortcomings. In this retrospective study, we found that the combination of the PI-RADS score and PSAD can achieve outstanding diagnostic performance of PCa. Patients with “PI-RADS score < 3 and PSAD < 0.3” may safely avoid biopsies and execute active surveillance, and patients with “PI-RADS score > 3 and PSAD ≥ 0.3” can also take a radical or systematic therapy without prostate biopsies into consideration. However, a study with prospective design is still needed to further confirm our findings in the future.

## Data availability statement

The raw data supporting the conclusions of this article will be made available by the authors, without undue reservation.

## Ethics statement

This study involving human participants was reviewed and approved by Ethics committee of The First Affiliated Hospital of USTC. The patients/participants provided their written informed consent to participate in this study.

## Author contributions

Study concept and design: JX and TT. Acquisition of data: CW, LY, and BZ. Analysis and interpretation of data: LY, DS, and BZ. Statistical analysis: CW, BW, and PZ. Drafting of the manuscript: CW. Review and supervision: JX and TT. All authors contributed to the article and approved the submitted version.

## Funding

The current study was partly supported by the Key Researchand Development Program of Anhui Province (No.1804h08020253 and 202004j07020022).

## Conflict of interest

The authors declare that the research was conducted in the absence of any commercial or financial relationships that could be construed as a potential conflict of interest.

## Publisher’s note

All claims expressed in this article are solely those of the authors and do not necessarily represent those of their affiliated organizations, or those of the publisher, the editors and the reviewers. Any product that may be evaluated in this article, or claim that may be made by its manufacturer, is not guaranteed or endorsed by the publisher.

## Supplementary material

Supplementary Figure 1 The representative images of mpMRI of six patients: **(A)** PI-RADS score 1: Normal peripheral zone, axial T2WI shows uniformly hyperintensive signal intensity, high *b*-value DWI and ADC also show no abnormality (white arrow); **(B)** PI-RADS score 2: Axial T2WI shows diffuse hypointensity with indistinct margin, high *b*-value DWI exhibits slightly hyperintense and indistinct hypointense on ADC (white arrow); **(C)** PI-RADS score 3: Axial T2WI exhibits non-circumscribed moderate hypointensity in left peripheral zone, DWI shows mildly hyperintense on high *b*-value and hypointense on ADC (white arrow); **(D)** PI-RADS score 4: Axial T2WI shows circumscribed, homogenous moderate hypointense focus confined in right peripheral zone with greatest dimension <1.5cm, DWI sees focal obviously hyperintense on high *b*-value and hypointense on ADC (white arrow); **(E)** PI-RADS score 5: Axial T2WI shows non-circumscribed, homogenous moderate hypointense focus confined with greatest dimension >1.5cm in left transition zone and peripheral zone. high *b*-value DWI shows focal markedly hyperintense and apparent hypointense on ADC (white arrow); **(F)** Measurement of prostate maximum diameters, green line: anteroposterior diameter, pink line: longitudinal diameter, brown line: transverse diameter.

Supplementary Figure 2 Spearman correlation analysis between clinical variables and biopsy results indicated PI-RADS score and PSAD were closely related to the detection of PCa and csPCa. *, P < 0.05; **, P <0.01; ***, P <0.001.
