# Supplementary figures and images for: Combination of PI-RADS score and PSAD can improve the diagnostic accuracy of prostate cancer and reduce unnecessary prostate biopsies
Source: Front Oncol. 2022 Nov 16;12:1024204. doi: 10.3389/fonc.2022.1024204 (PMC9709422; doi:10.3389/fonc.2022.1024204)

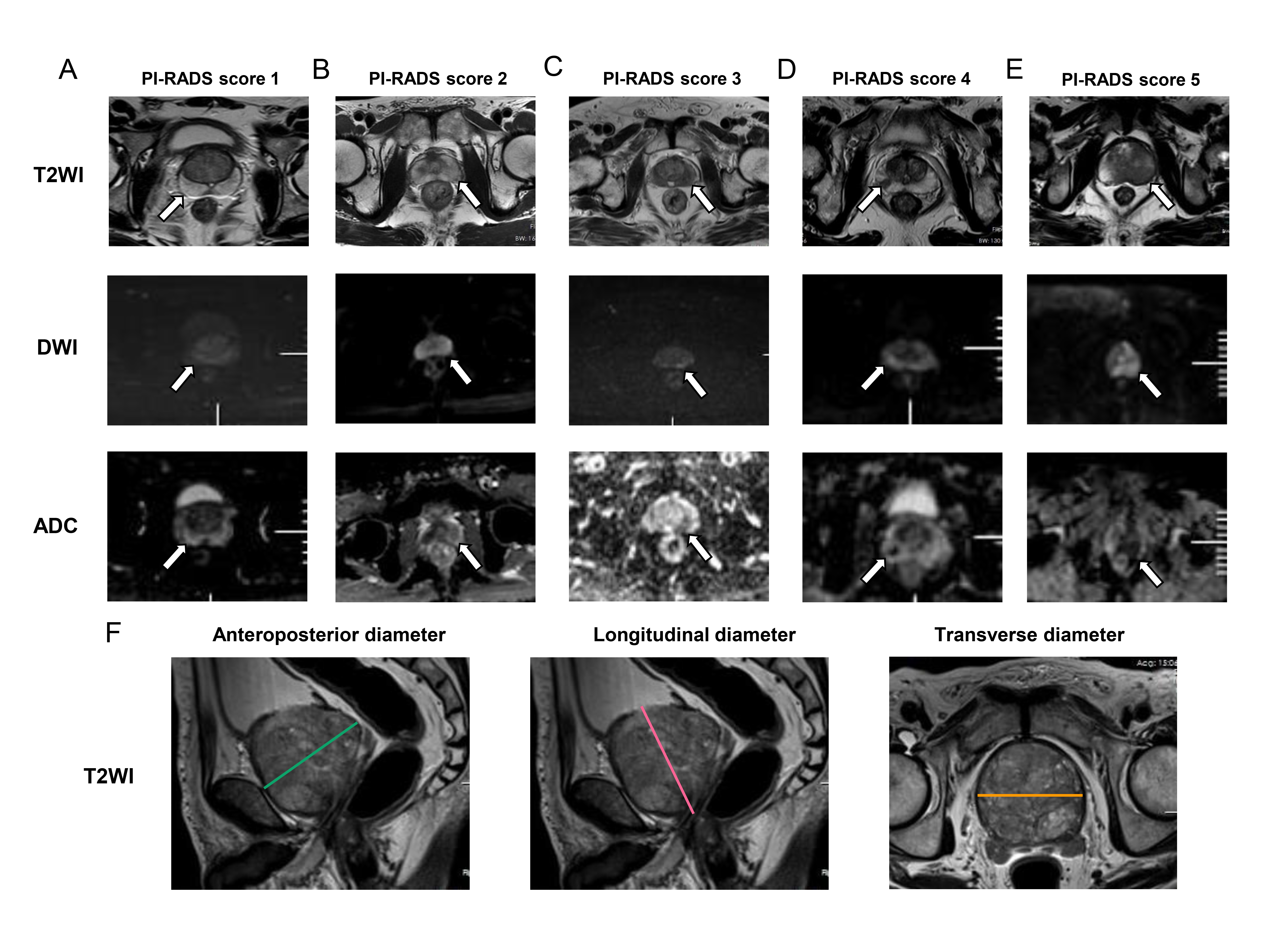

Supplement: Supplementary Figure 1 — The representative images of mpMRI of six patients: (A) PI-RADS score 1: Normal peripheral zone, axial T2WI shows uniformly hyperintensive signal intensity, high b-value DWI and ADC also show no abnormality (white arrow); (B) PI-RADS score 2: Axial T2WI shows diffuse hypointensity with indistinct margin, high b-value DWI exhibits slightly hyperintense and indistinct hypointense on ADC (white arrow); (C) PI-RADS score 3: Axial T2WI exhibits non-circumscribed moderate hypointensity in left peripheral zone, DWI shows mildly hyperintense on high b-value and hypointense on ADC (white arrow); (D) PI-RADS score 4: Axial T2WI shows circumscribed, homogenous moderate hypointense focus confined in right peripheral zone with greatest dimension <1.5cm, DWI sees focal obviously hyperintense on high b-value and hypointense on ADC (white arrow); (E) PI-RADS score 5: Axial T2WI shows non-circumscribed, homogenous moderate hypointense focus confined with greatest dimension >1.5cm in left transition zone and peripheral zone. high b-value DWI shows focal markedly hyperintense and apparent hypointense on ADC (white arrow); (F) Measurement of prostate maximum diameters, green line: anteroposterior diameter, pink line: longitudinal diameter, brown line: transverse diameter. [file Image_1.tif]

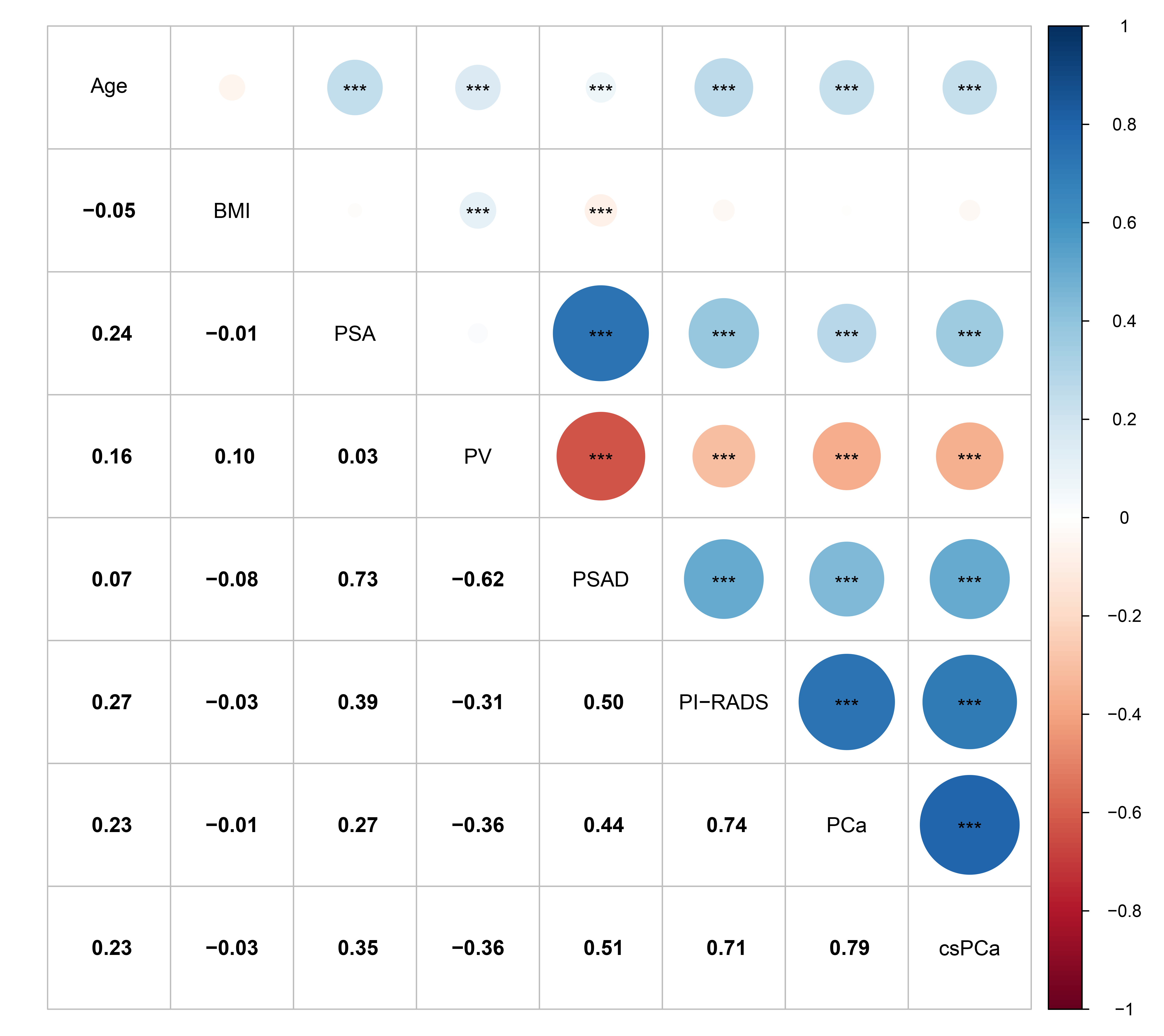

Supplement: Supplementary Figure 2 — Spearman correlation analysis between clinical variables and biopsy results indicated PI-RADS score and PSAD were closely related to the detection of PCa and csPCa. *, P < 0.05; **, P <0.01; ***, P <0.001. [file Image_2.tif]
