# Supplementary material for: Combination of PI-RADS score and PSAD can improve the diagnostic accuracy of prostate cancer and reduce unnecessary prostate biopsies
Source: Front Oncol. 2022 Nov 16;12:1024204. doi: 10.3389/fonc.2022.1024204 (PMC9709422; doi:10.3389/fonc.2022.1024204)
Supplement: Supplementary file 3 [file Table_1.docx]

| Parameters | Total patients | Non-PCa | Total PCa | cisPCa | csPCa |
| --- | --- | --- | --- | --- | --- |
|  | Median (IQR) | Median (IQR) | Median (IQR) | Median (IQR) | Median (IQR) |
| Age (years) | 69.00 (63.00-75.00) | 68.00 (61.00-73.00) | 72.00 (66.00-77.00) | 70.00 (65.00-76.00) | 73.00 (67.00-78.00) |
| BMI (kg/m^2^) | 23.77 (21.80-25.50) | 23.77 (21.90-25.50) | 23.78 (21.60-25.49) | 23.89 (22.01-25.57) | 23.71 (21.42-25.45) |
| PSA (ng/ml) | 13.83 (9.36-21.61) | 12.24 (8.74-18.32) | 17.79 (10.79-31.95) | 11.45 (8.43-18.72) | 20.43 (13.28-40.48) |
| PV (ml) | 47.62 (32.18-67.78) | 56.87 (39.95-79.63) | 36.95 (26.85-54.44) | 43.93 (23.88-64.20) | 35.92 (25.82-47.96) |
| PSAD (ng/ml^2^) | 0.30 (0.16-0.52) | 0.22 (0.14-0.36) | 0.48 (0.27-0.96) | 0.25 (0.16-0.45) | 0.60 (0.36-1.14) |
|  | Case (%) | Case (%) | Case (%) | Case (%) | Case (%) |
| Total | 833 (100.0%) | 497 (100%) | 336 (100%) | 88 (100%) | 248 (100%) |
| PI-RADS score |  |  |  |  |  |
| 1 | 48 (5.8%) | 46 (9.3%) | 2 (0.6%) | 2 (2.3%) | 0 (0.0%) |
| 2 | 384 (46.1%) | 354 (71.2%) | 30 (8.9%) | 21 (23.9%) | 9 (3.6%) |
| 3 | 123 (14.8%) | 68 (13.7%) | 55 (16.4%) | 25 (28.4%) | 30 (12.1%) |
| 4 | 128 (15.4%) | 21 (4.2%) | 107 (31.8%) | 29 (33.0%) | 78 (31.5%) |
| 5 | 150 (18.0%) | 8 (1.6%) | 142 (42.3%) | 11(12.5%) | 131 (52.8%) |
| Gleason score |  |  |  |  |  |
| No cancer | 497 (59.7%) | 497 (100%) |  |  |  |
| 6 | 88 (10.6%) |  | 88 (26.2%) | 88 (100%) |  |
| 7 | 108 (13.0%) |  | 108 (32.1%) |  | 108 (43.5%) |
| 8 | 82 (9.8%) |  | 82 (24.4%) |  | 82 (33.1%) |
| 9 | 40 (4.8%) |  | 40 (11.9%) |  | 40 (16.1%) |
| 10 | 18 (2.2%) |  | 18 (5.4%) |  | 18 (7.3%) |

**TABLE S1** Demographic characteristics of all the biopsy-naïve patients

*PCa, prostate cancer;* *csPCa, clinically significant prostate cancer; cisPCa, clinically insignificant prostate cancer; IQR, interquartile range; BMI, body mass index; PSA, prostate-specific antigen; PV, prostate volume; PSAD**, prostate-specific antigen density; PI-RADS**, prostate imaging-reporting and data system.*

**TABLE S2** PCa detection rate stratified by PI-RADS and PSAD

| Grouping variables | | PI-RADS score | | | | | Total |
| --- | --- | --- | --- | --- | --- | --- | --- |
|  |  | 1 | 2 | 3 | 4 | 5 |  |
| PSAD  (ng/ml^2^) | <0.15 | 0/22(0.0%) | 10/109(9.2%) | 5/17(29.4%) | 13/16(81.3%) | 1/3(33.3%) | 29/167(17.4%) |
|  | 0.15-0.30 | 1/12(8.3%) | 9/148(6.1%) | 21/53(39.6%) | 17/25(68.0%) | 18/18(100.0%) | 66/256(25.8%) |
|  | 0.30-0.60 | 1/14(7.1%) | 7/93(7.5%) | 18/35(51.4%) | 43/51(84.3%) | 34/37(91.9%) | 103/230(44.8%) |
|  | 0.60-0.90 | 0/0(NA) | 3/20(15.0%) | 5/10(50.0%) | 16/18(88.9%) | 25/26(96.2%) | 49/74(66.2%) |
|  | ≥0.9 | 0/0(NA) | 1/14(7.1%) | 6/8(75.0%) | 18/18(100.0%) | 64/66(97.0%) | 89/106(84.0%) |
|  | Total | 2/48(4.2%) | 30/384(7.8%) | 55/123(44.7%) | 107/128 (83.6%) | 142/150(94.7%) | 336/833(40.3%) |

*PCa, prostate cancer; PSAD, prostate-specific antigen density; PI-RADS, prostate imaging-reporting and data system.*

**TABLE S3** csPCa detection rate stratified by PI-RADS score and PSAD

| Grouping variables | | PI-RADS score | | | | | Total |
| --- | --- | --- | --- | --- | --- | --- | --- |
|  |  | 1 | 2 | 3 | 4 | 5 |  |
| PSAD  (ng/ml^2^) | <0.15 | 0/22(0.0%) | 1/109(0.9%) | 2/17(11.8%) | 7/16(43.8%) | 1/3(33.3%) | 11/167(6.6%) |
|  | 0.15-0.30 | 0/12(0.0%) | 1/148(0.7%) | 10/53(18.9%) | 9/25(36.0%) | 14/18(77.8%) | 34/256(13.3%) |
|  | 0.30-0.60 | 0/14(0.0%) | 4/93(4.3%) | 9/35(25.7%) | 34/51(66.7%) | 31/37(83.8%) | 78/230(33.9%) |
|  | 0.60-0.90 | 0/0(NA) | 2/20(10.0%) | 3/10(30.0%) | 12/18(66.7%) | 23/26(88.5%) | 40/74(54.1%) |
|  | ≥0.9 | 0/0(NA) | 1/14(7.1%) | 6/8(75.0%) | 16/18(88.9%) | 62/66(93.9%) | 85/106(80.2%) |
|  | Total | 0/48(0.0%) | 9/384(2.3%) | 30/123(24.4%) | 78/128(60.9%) | 131/150(87.3%) | 248/833(29.8%) |

*csPCa, clinically significant prostate cancer; PSAD, prostate-specific antigen density; PI-RADS, prostate imaging-reporting and data system.*

**TABLE S4** Prostate cancer detection rate of different group stratified by PI-RADS score and PSAD

|  | Total | ^*^Group 1 | ^^^Group 2 | ^#^Group 3 |
| --- | --- | --- | --- | --- |
| Data of our own cohort | | | | |
| All patients | 833 (100%) | 291 (100%) | 326 (100%) | 216 (100%) |
| Non cancer | 497 (59.7%) | 271 (93.1%) | 210 (64.4%) | 16 (7.4%) |
| Total PCa | 336 (40.3%) | 20 (6.9%) | 116 (35.6%) | 200 (92.6%) |
| cisPCa | 88 (10.6%) | 18 (6.2%) | 48 (14.7%) | 22 (10.2%) |
| csPCa | 248 (29.8%) | 2 (0.7%) | 68 (20.9%) | 178 (82.4%) |
| External validation dataset one | | | | |
| All patients | 342 (100%) | 104 (100%) | 146 (100%) | 92 (100%) |
| Non cancer | 205 (59.9%) | 94(90.4%) | 103 (70.5%) | 8 (8.7%) |
| Total PCa | 137 (40.1%) | 10 (9.6%) | 43 (29.5%) | 84 (91.3%) |
| cisPCa | 20 (5.9%) | 3 (2.9%) | 12 (8.3%) | 5 (5.4%) |
| csPCa | 117 (34.2%) | 7 (6.7%) | 31 (21.2%) | 79 (85.9%) |
| External validation dataset two | | | | |
| All patients | 385 (100%) | 101 (100%) | 186 (100%) | 98 (100%) |
| Non cancer | 188 (48.8%) | 94 (93.1%) | 87 (46.8%) | 7 (7.1%) |
| Total PCa | 197 (51.2%) | 7 (6.9%) | 99 (53.2%) | 91 (92.9%) |
| cisPCa | 35 (9.1%) | 3 (3.0%) | 20 (10.7%) | 12 (12.3%) |
| csPCa | 162 (42.1%) | 4 (3.9%) | 79 (42.5%) | 79 (80.6%) |

*PCa, prostate cancer; cisPCa, clinically insignificant prostate cancer; csPCa, clinically significant prostate cancer; PSAD, prostate-specific antigen density; PI-RADS, prostate imaging-reporting and data system. ^*^Group 1 patients with PI-RADS score＜3 and PSAD＜0.3; ^^^Group 2 patients with ‘PI-RADS score≥3 and PSAD＜0.3’ or ‘PI-RADS score＜3 and PSAD≥0.3’; ^#^Group 3 patients with PI-RADS score＞3 and PSAD≥0.3.*
